# Supplementary material for: A delay in sampling information from temporally autocorrelated visual stimuli
Source: Nat Commun. 2020 Apr 15;11:1852. doi: 10.1038/s41467-020-15675-1 (PMC7160117; doi:10.1038/s41467-020-15675-1)
Supplement: Supplementary file 1 — Supplementary Information [file 41467_2020_15675_MOESM1_ESM.pdf]

Supplementary information for "A delay in sampling information from temporally autocorrelated visual stimuli"

Callahan-Flintoft, C., Holcombe, A. O., & Wyble, B.

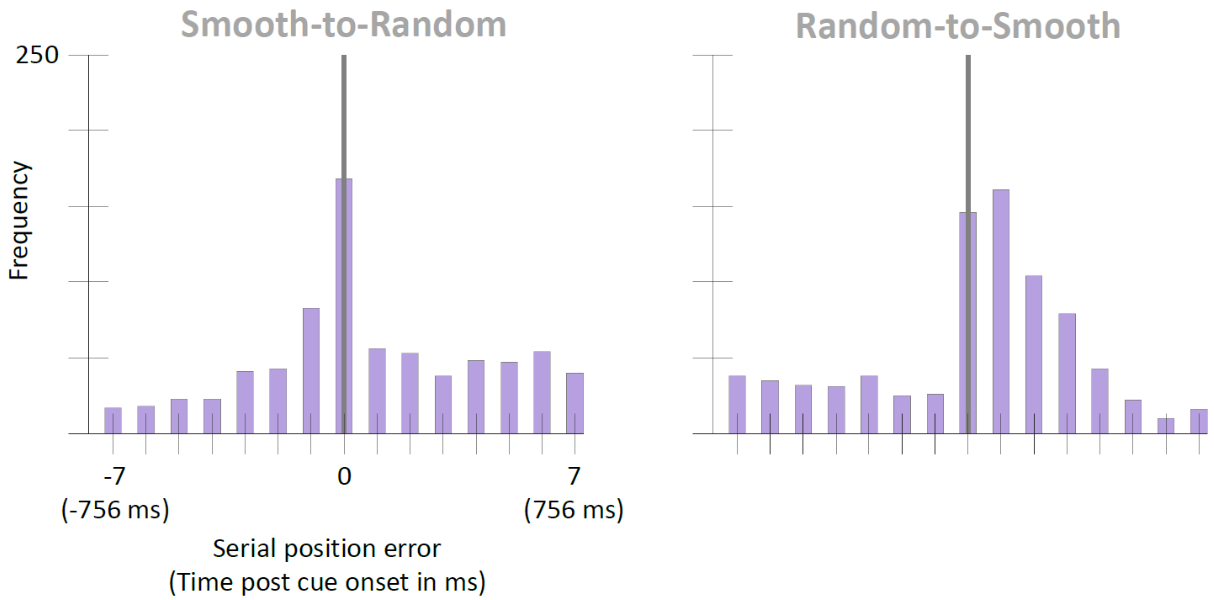

Supplementary Figure 1: Serial position errors of Experiment 3 comparing reports to actual colors presented to participants (i.e. smooth and random presentation before or after the cue depending on condition). The grey vertical line marks the position of the cued color.

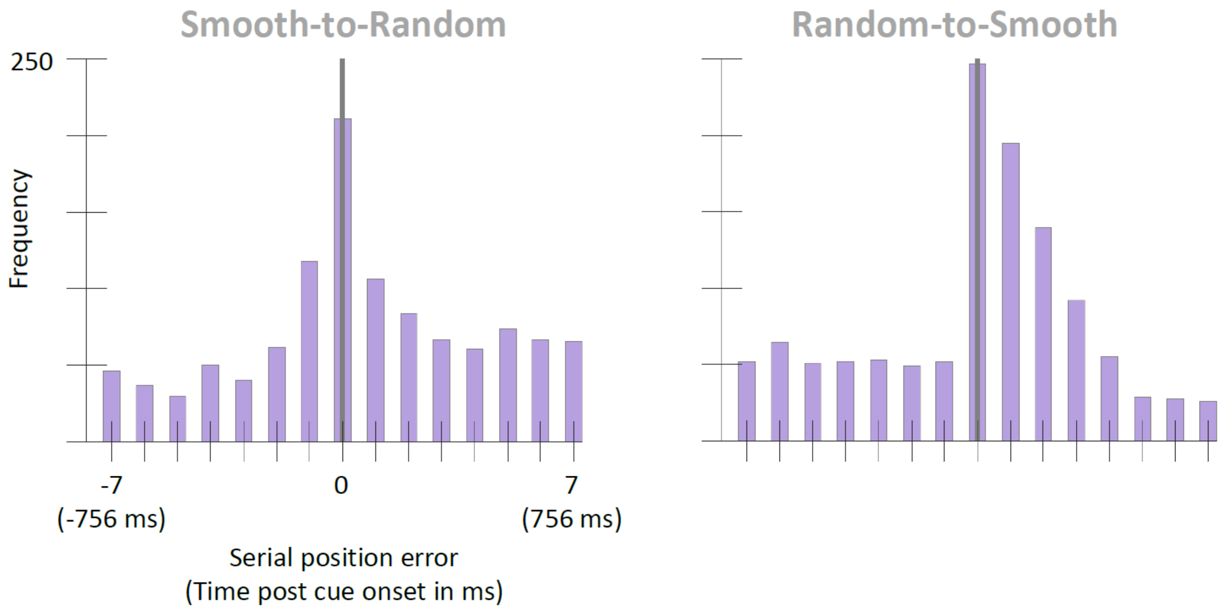

Supplementary Figure 2: Serial position errors of Experiment 3's replication study, comparing reports to actual colors presented to participants (i.e. smooth and random presentation before or after the cue depending on condition). The grey vertical line marks the position of the cued color.

		Mean Difference (ms)	*p*-value	95% CI of mean difference (ms)
Exp 1	< 6°	91	<.001	[54, 129]
	< 16°	110	<.001	[76, 135]
	No constraints	114	<.001	[81, 144]
Exp 1 (rep)	< 6°	104	<.001	[66, 142]
	< 16°	113	<.001	[78, 137]
	No constraints	111	<.001	[80, 142]

Supplementary Table 1. Statistics for Experiment 1 & Experiment 1's replication for 3 different levels of constraints. In "No constraint", all trial were excluded except those that were tied for minimum distance between two colors presented colors. The "< 16°" version is what is presented in the paper and the "< 6°" version is where all trials outside of 6 degrees of the presented colors were excluded. The same two-sided, permutation analysis described in the main text was used here. No correction for multiple comparisons has been implemented. These results have been included not as replications but as a demonstration that the significant results presented in the paper are not contingent on the specific constraints chosen.

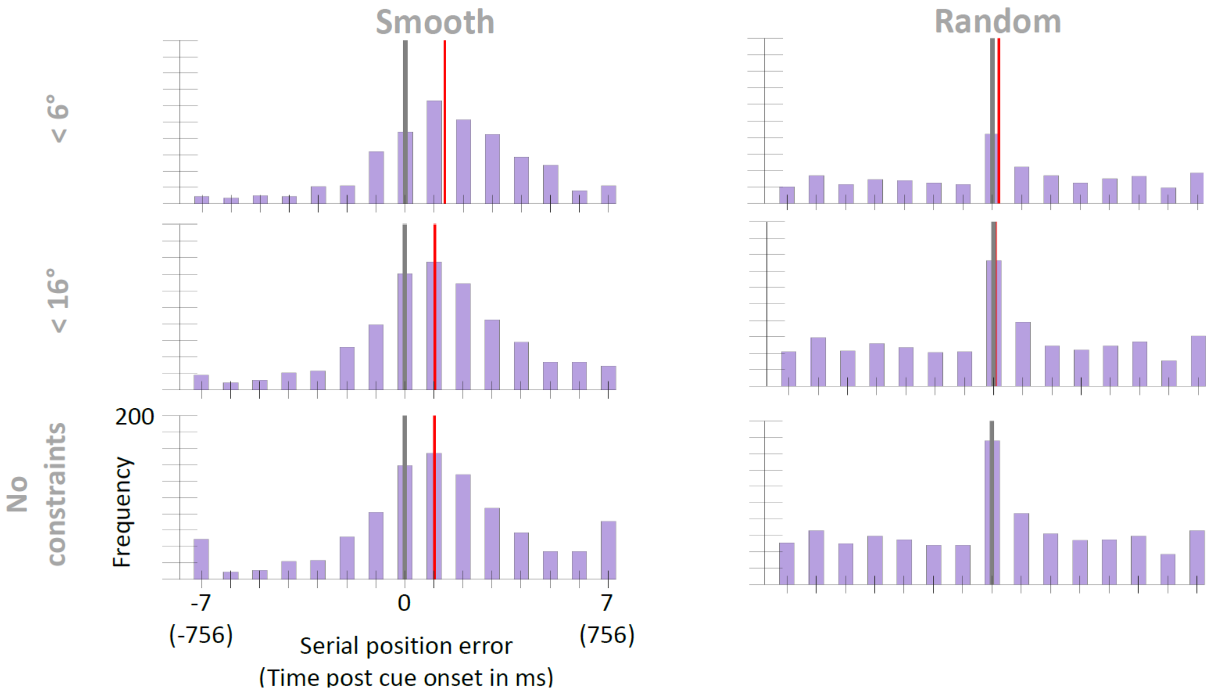

Supplementary Figure 3: Serial position error histograms aggregated across 25 participants in Experiment 1. Top: data analyzed using a threshold of 6 degrees, such that trials with a minimum difference exceeding 6 degrees from the colors presented 7 positions before and after the cue were excluded. Middle: data analyzed using a threshold of 16 degrees (presented in paper). Bottom: data analyzed without constraints, only excluding those trials that had a tied minimum difference between 2 presented colors. The grey vertical line marks the position of the cued color and the red line marks the condition mean.

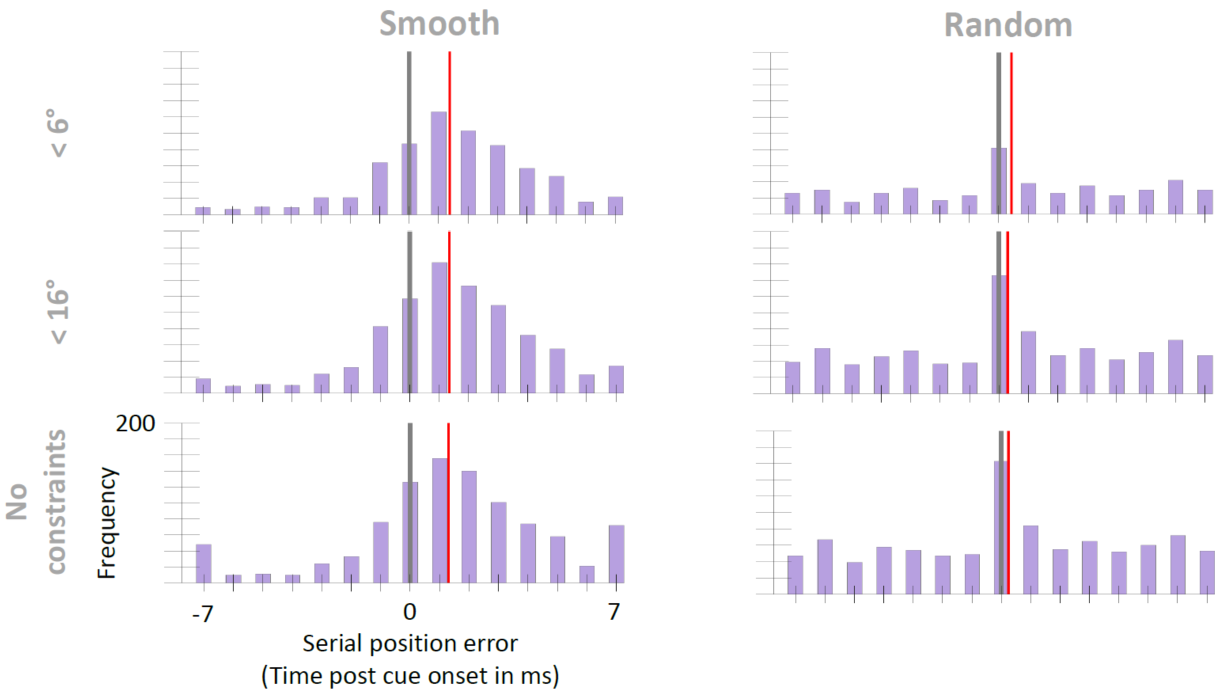

Supplementary Figure 4: Serial position error histograms aggregated across 25 participants in Experiment 1's replication study. Top: data analyzed using a threshold of 6 degrees, such that trials with a minimum difference exceeding 6 degrees from the colors presented 7 positions before and after the cue were excluded. Middle: data analyzed using a threshold of 16 degrees (presented in paper). Bottom: data analyzed without constraints, only excluding those trials that had a tied minimum difference between 2 presented colors. The grey vertical line marks the position of the cued color and the red line marks the condition mean.

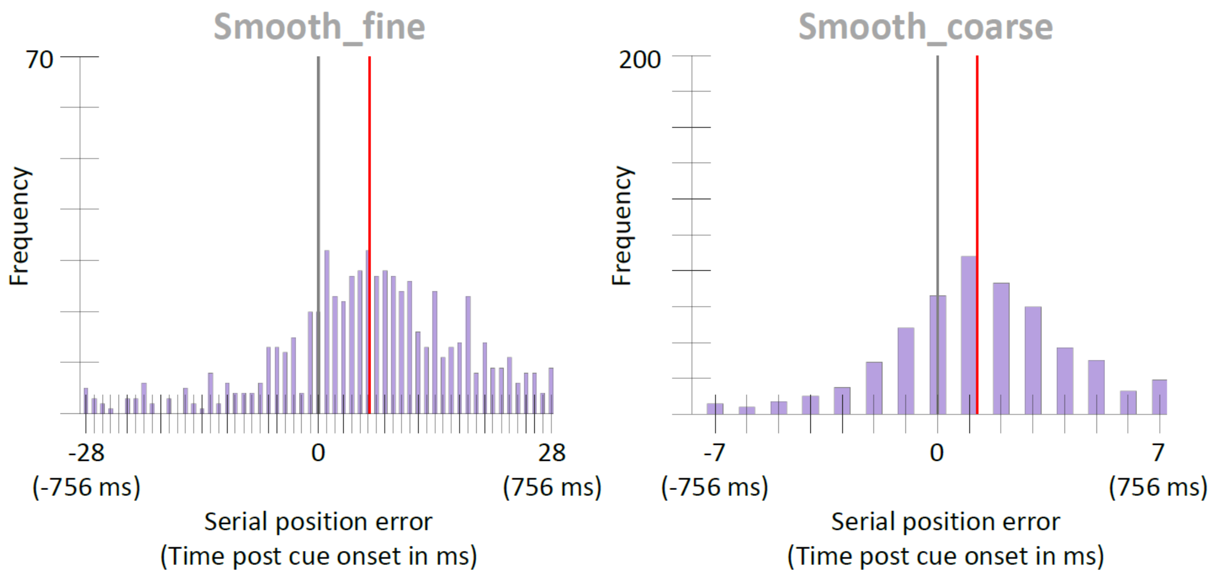

Supplementary Figure 5: Serial position error histograms aggregated across 25 participants in Experiment 2, re-analyzed with a lower exclusion criteria. Here, all trials where the reported color value was over 4° from all presented colors were excluded from analysis. This resulted in average of 18 trials (SD = 4) excluded from the Smooth_coarse condition and 12 trials (SD = 4) excluded from the Smooth_fine condition. Using a 2-sided permutation analysis (described in the Analysis section of the main text), a significant delay in selection latency of 31 ms in the Smooth_fine condition compared to the Smooth_coarse condition, p = .04, 95% CI [4, 59]. The grey vertical line marks the position of the cued color and the red line marks the condition mean.

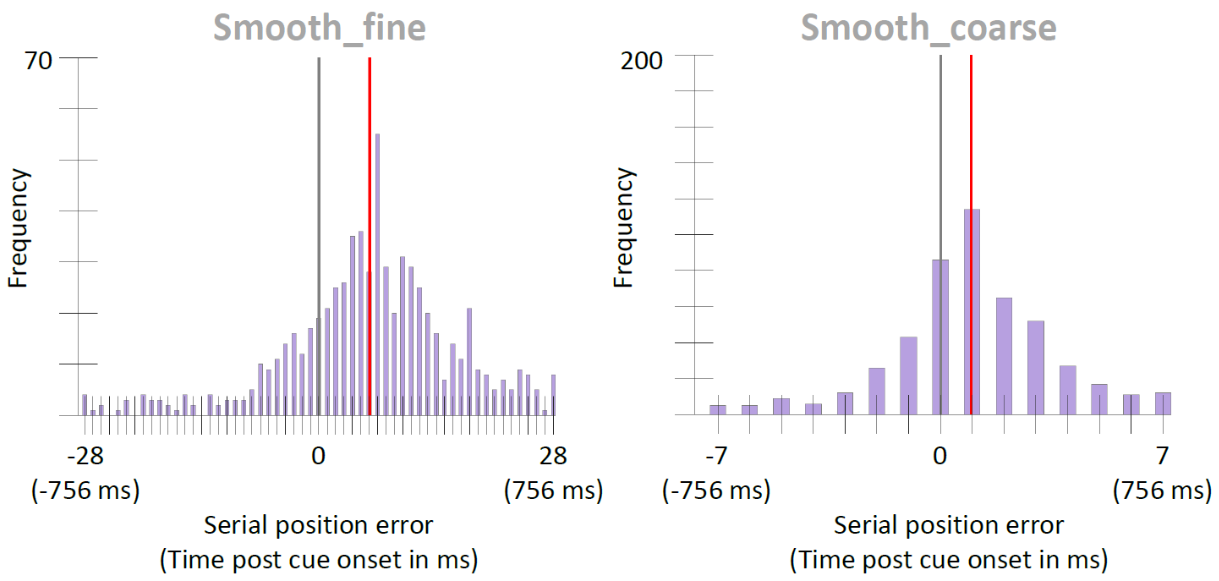

Supplementary Figure 6: Serial position error histograms aggregated across 25 participants in Experiment 2's replication study data, excluding all trials where the reported color value was over 4° from all presented colors. Nineteen trials (*SD* = 4) were excluded from the Smooth_coarse condition and 12 trials (*SD* = 3) were excluded from the Smooth_fine condition. Using a 2-sided permutation analysis (described in the Analysis section of the main text), a significant delay in selection latency of 59 ms in the Smooth_fine condition compared to the Smooth_coarse condition was found, $p = .001$, 95% CI [32, 84]. The grey vertical line marks the position of the cued color and the red line marks the condition mean.

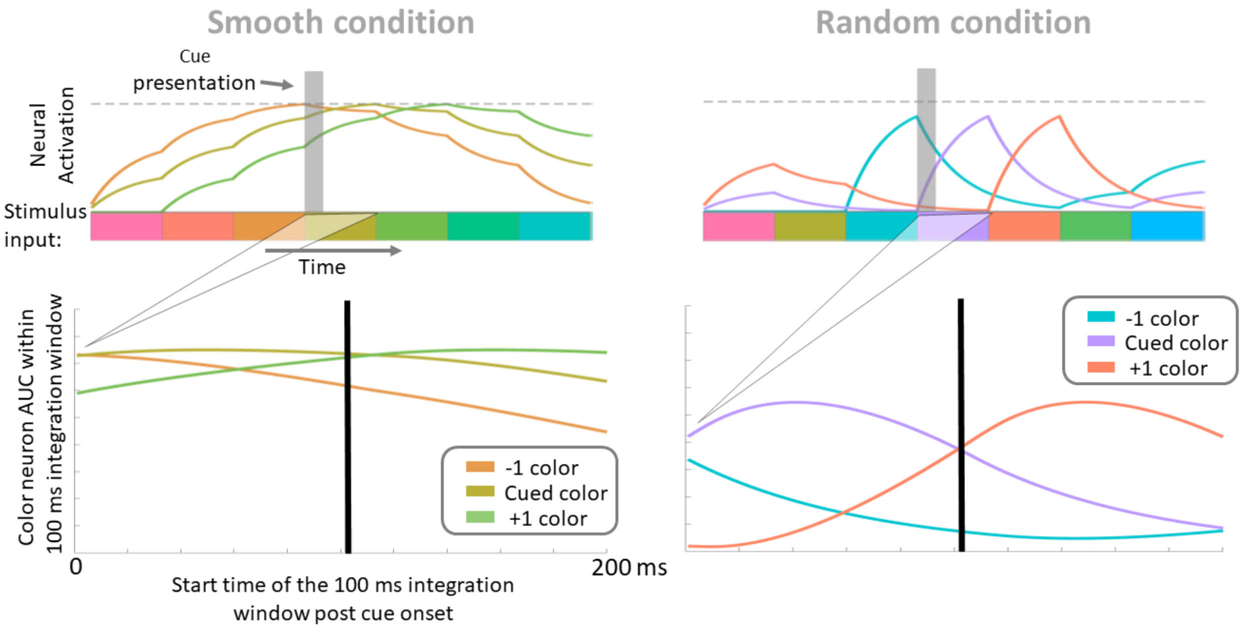

Supplementary Figure 7: Model simulations of neural activation which shows that a simple, fixed integration interval theory is unable to account for the results seen in Experiment 1. Top: The neural activation of the neuron most responsive to the cued color and those most response of the color presented before the cued color (-1 color) and after the cued color (+1) for both Smooth (left) and Random (right) presentation. The transparent vertical grey bar in both graphs represents the duration of the cue's presentation. Bottom: The calculated area under the curve (AUC) of each color neuron within a 100 ms window. The transparent triangle relates the first 100 ms window after the cue onset to the first data points of the bottom graphs. This calculation was repeated 200 times, each time moving the integration window one millisecond later in order to test whether there was a single placement of the integration window that would yield the highest AUC for the +1 color in the Smooth condition while producing the highest AUC of the cued color in the Random condition (effectively simulating the shifted mean selection time in the Smooth condition seen in Experiment 1). Using the Random data as a benchmark, an upper bound is set on how late the integration window can be by marking the point at which AUC of the +1 color neuron within the window would exceed the cued color neuron's AUC. Applying the same boundary to the Smooth condition (denoted as a black vertical line in both bottom graphs), we see that no matter where the window is placed prior to that boundary, the cued color neuron still shows the strongest activation. Thus, the fixed integration window, as implemented here, is unable to account for the mean shift between conditions seen in the experimental data. There of course may be other ways in which to implement a fixed integration window in order to simulate the data presented in Experiments 1 & 2, however this would require further development and possible the addition of other neural mechanisms.

Supplementary Notes

This experiment was conducted to test whether the duration of the cue would have an effect on the selection latency of a feature. In previous unpublished pilot experiments associated with the experiments described in Holcombe & Cavanagh (2008)[1], attentional selection from temporally autocorrelated stimuli seemed more effective with a longer cue. As a brief cue is not well matched to the poor temporal resolution of attention[2], with a brief cue it is possible that sampling must occur via more endogenous attention, resulting in longer latency. This suggests that with a longer cue, there might be a shorter sampling latency.

The attentional drag theory, in contrast, attributes long selection latencies to the temporal autocorrelation of smoothly changing stimuli and does not suggest there should be an effect of duration of the cue. To test this, two conditions were compared, varying the duration of cue presentation.

Method

Participants. A sample of 24 undergraduates age 18-23 with normal to correct to normal vision were used for this experiment with the same recruitment and consent procedure as outlined in all of the previous experiments.

Stimuli & Apparatus. The same stimuli used in the first 3 experiments were used in this experiment.

Procedure. In this experiment participants again maintained fixation on a cross in the middle of the screen and monitored two changing colored disks. The colors changed in the same fashion as the Smooth condition of Experiment 1, stepping 16 degrees around a color ring every 108 ms. Participants were told to report the color of the disk at the time of the cue. The same cueing and reporting method used in the previous 3 experiments were used here.

In this experiment there were two conditions. The Short condition was identical to the Smooth condition of Experiment 1 where the cue was presented only for the first 27 ms of the cued color's presentation. In the Long condition the cue was presented for 108 ms (the same duration as the cued color).

Results

The same permutation analysis and bootstrap method described in Experiment 1 were used here. There was a non-significant delay in the selection latency for the Long Cue condition compared to the Short Cue condition (24 ms), p = .08, 95% CI [-3, 50]. If anything, the data suggest that the latency is longer in the Long condition, which is contrary to our concern that the Long condition might conceivably reduce or eliminate the attentional drag effect, if the effect reflected ineffectiveness of attention specific to an overly brief cue.

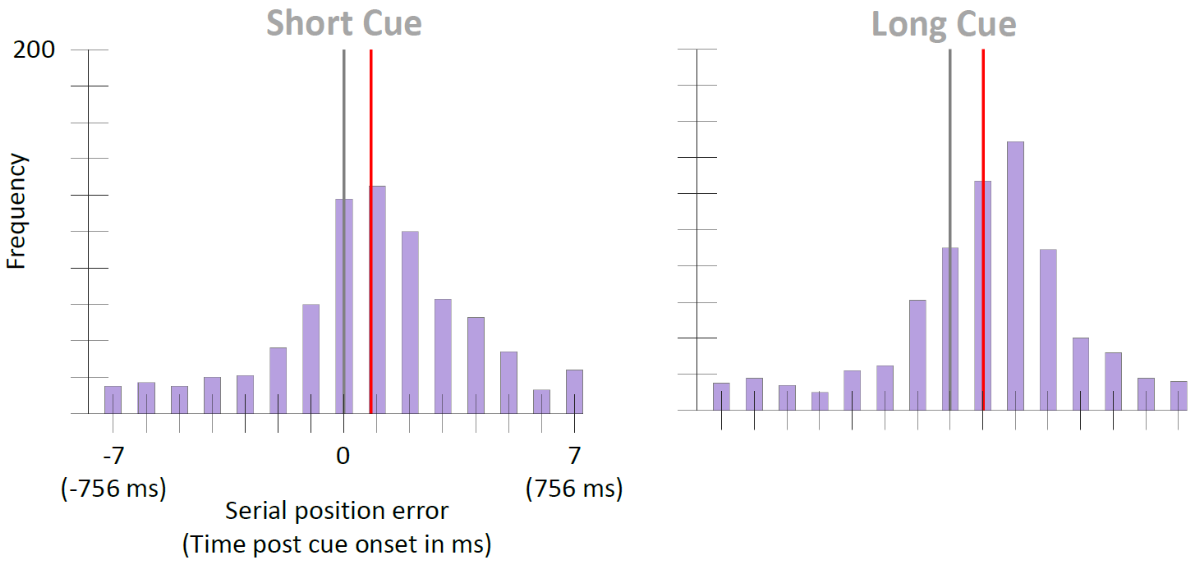

Supplementary Figure 8: Serial position error histograms aggregated across 25 participants in the experiment described in the supplementary notes. The grey vertical line marks the position of the cued color and the red line marks the condition mean.

Supplementary References

1 Holcombe, A. O. & Cavanagh, P. Independent, synchronous access to color and motion features. *Cognition* **107**, 552-580, doi:10.1016/j.cognition.2007.11.006 (2008).

2 Holcombe, A. O. Seeing slow and seeing fast: two limits on perception. *Trends in cognitive sciences* **13**, 216-221, doi:10.1016/j.tics.2009.02.005 (2009).
