# Supplementary material for: A delay in sampling information from temporally autocorrelated visual stimuli
Source: Nat Commun. 2020 Apr 15;11:1852. doi: 10.1038/s41467-020-15675-1 (PMC7160117; doi:10.1038/s41467-020-15675-1)
Supplement: Supplementary file 3 — Reporting Summary [file 41467_2020_15675_MOESM3_ESM.pdf]

nature research

Corresponding author(s): Chloe Callahan-Flintoft

Last updated by author(s): Mar 2, 2020

Reporting Summary

Nature Research wishes to improve the reproducibility of the work that we publish. This form provides structure for consistency and transparency in reporting. For further information on Nature Research policies, see Authors & Referees and the Editorial Policy Checklist.

Statistics

For all statistical analyses, confirm that the following items are present in the figure legend, table legend, main text, or Methods section.

n/a	Confirmed	
☐	☒	The exact sample size (*n*) for each experimental group/condition, given as a discrete number and unit of measurement
☐	☒	A statement on whether measurements were taken from distinct samples or whether the same sample was measured repeatedly
☐	☒	The statistical test(s) used AND whether they are one- or two-sided<br>*Only common tests should be described solely by name; describe more complex techniques in the Methods section.*
☒	☐	A description of all covariates tested
☐	☒	A description of any assumptions or corrections, such as tests of normality and adjustment for multiple comparisons
☐	☒	A full description of the statistical parameters including central tendency (e.g. means) or other basic estimates (e.g. regression coefficient) AND variation (e.g. standard deviation) or associated estimates of uncertainty (e.g. confidence intervals)
☐	☒	For null hypothesis testing, the test statistic (e.g. F, t, r) with confidence intervals, effect sizes, degrees of freedom and P value noted<br>*Give P values as exact values whenever suitable.*
☒	☐	For Bayesian analysis, information on the choice of priors and Markov chain Monte Carlo settings
☒	☐	For hierarchical and complex designs, identification of the appropriate level for tests and full reporting of outcomes
☐	☒	Estimates of effect sizes (e.g. Cohen's *d*, Pearson's *r*), indicating how they were calculated

Our web collection on statistics for biologists contains articles on many of the points above.

Software and code

Policy information about availability of computer code

Data collection	Psychtoolbox-3 functions and the Stream toolbox (https://osf.io/tdvxm/wiki/home/) were run using MATLAB 2017 to collect this data. All the code needed for running the paradigms can be found at https://osf.io/hujwb/
Data analysis	The analysis code used in these experiments was custom built and is available at https://osf.io/hujwb/

For manuscripts utilizing custom algorithms or software that are central to the research but not yet described in published literature, software must be made available to editors/reviewers. We strongly encourage code deposition in a community repository (e.g. GitHub). See the Nature Research guidelines for submitting code & software for further information.

Data

Policy information about availability of data

All manuscripts must include a data availability statement. This statement should provide the following information, where applicable:

- Accession codes, unique identifiers, or web links for publicly available datasets
- A list of figures that have associated raw data
- A description of any restrictions on data availability

The datasets generated and analyzed during the current study are available on the Open Science Framework (OSF) (https://osf.io/hujwb/).

Field-specific reporting

Please select the one below that is the best fit for your research. If you are not sure, read the appropriate sections before making your selection.

☐ Life sciences ☒ Behavioural & social sciences ☐ Ecological, evolutionary & environmental sciences

October 2018

Behavioural & social sciences study design

All studies must disclose on these points even when the disclosure is negative.

Study description	This was a quantitative, within-subjects experimental design.
Research sample	Participants were 18-23 years old undergraduates from the Pennsylvania State University taking introductory psychology courses. These students elected to participate in this study using the university subject pool in exchange for course credit.
Sampling strategy	The sampling procedure was convenience sampling. A posting was put up on the university subject pool website and students volunteered to participate for course credit. A sample size of 25 was selected based on pilot work. The results were then verified through a replication study for each experiment.
Data collection	All data was collected and stored on experiment computers running Windows XP. The researcher welcomed participants as they arrived and gave them informed consent forms. Once the consent forms were signed the researcher gave the participant a participant ID number and showed them to the computer they would use. The researcher sat in the same room but behind a partition while the participants completed the experiment. At the end of the experiment the participant told the researcher they were done and received a debriefing sheet explaining the purpose of the study. All of the task instruction were delivered by the computer, contained in the paradigm code.
Timing	All of the data was collected between August 2017 and September 2018
Data exclusions	Trials were excluded if the reported value was an equal distance from the two closest colors presented (i.e. a tie). Trials were also excluded if this minimum difference between the reported color and the color of the closest matching stimulus was greater than 16 degrees of the 360 degrees of the color circle. These criteria as well as the summary statistics for the number of trials excluded per participant is included in the manuscript.
Non-participation	No participants dropped out of this study.
Randomization	There was only one group in this experiment. All participants saw trials of both conditions intermixed within block. The order the trials were presented was randomized using a random number generator.

Reporting for specific materials, systems and methods

We require information from authors about some types of materials, experimental systems and methods used in many studies. Here, indicate whether each material, system or method listed is relevant to your study. If you are not sure if a list item applies to your research, read the appropriate section before selecting a response.

Materials & experimental systems

n/a	Involved in the study	
☒	☐	Antibodies
☒	☐	Eukaryotic cell lines
☒	☐	Palaeontology
☒	☐	Animals and other organisms
☐	☒	Human research participants
☒	☐	Clinical data

Methods

n/a	Involved in the study	
☒	☐	ChIP-seq
☒	☐	Flow cytometry
☒	☐	MRI-based neuroimaging

Human research participants

Policy information about studies involving human research participants

Population characteristics	Participants were undergraduates, ages 18 to 23, enrolled at the Pennsylvania State University.
Recruitment	The Pennsylvania State University subject pool was used to recruit participants. All participants in the presented work participated in this study for course credit.
Ethics oversight	The Internal Review Board of the Pennsylvania State University oversaw this study.

Note that full information on the approval of the study protocol must also be provided in the manuscript.
